# The User-Centered Design as Novel Perspective for Evaluating the Usability of BCI-Controlled Applications

**DOI:** 10.1371/journal.pone.0112392

**Published:** 2014-12-03

**Authors:** Andrea Kübler, Elisa M. Holz, Angela Riccio, Claudia Zickler, Tobias Kaufmann, Sonja C. Kleih, Pit Staiger-Sälzer, Lorenzo Desideri, Evert-Jan Hoogerwerf, Donatella Mattia

**Affiliations:** 1 Institute of Psychology, University of Würzburg, Würzburg, Germany; 2 Laboratory of Neuroelectrical Imaging and BCI, Fondazione Santa Lucia, IRCCS, Rome Italy; 3 Institute of Medical Psychology and Behavioural Neurobiology, University of Tübingen, Tübingen, Germany; 4 Psychosis Research Center, Institute of Clinical Medicine, University of Oslo, Oslo, Norway; 5 Beratungsstelle für Unterstützte Kommunikation (BUK), Diakonie Bad-Kreuznach, Bad Kreuznach, Germany; 6 AIAS Bologna onlus/Ausilioteca, Bologna, Italy; University of Perugia, Italy

## Abstract

Albeit research on brain-computer interfaces (BCI) for controlling applications has expanded tremendously, we still face a translational gap when bringing BCI to end-users. To bridge this gap, we adapted the user-centered design (UCD) to BCI research and development which implies a shift from focusing on single aspects, such as accuracy and information transfer rate (ITR), to a more holistic user experience. The UCD implements an iterative process between end-users and developers based on a valid evaluation procedure. Within the UCD framework usability of a device can be defined with regard to its effectiveness, efficiency, and satisfaction. We operationalized these aspects to evaluate BCI-controlled applications. Effectiveness was regarded equivalent to accuracy of selections and efficiency to the amount of information transferred per time unit and the effort invested (workload). Satisfaction was assessed with questionnaires and visual-analogue scales. These metrics have been successfully applied to several BCI-controlled applications for communication and entertainment, which were evaluated by end-users with severe motor impairment. Results of four studies, involving a total of N = 19 end-users revealed: effectiveness was moderate to high; efficiency in terms of ITR was low to high and workload low to medium; depending on the match between user and technology, and type of application satisfaction was moderate to high. The here suggested evaluation metrics within the framework of the UCD proved to be an applicable and informative approach to evaluate BCI controlled applications, and end-users with severe impairment and in the locked-in state were able to participate in this process.

## Introduction

While in 1999, when the first study about BCI-based communication by two locked-in patients was published [1], it needed to be demonstrated that muscle independent communication was at all possible; this by now has been demonstrated with several severely impaired individuals [2]–[7]. Yet, studies involving end-users with severe disability are still sparse owing to difficulties with access to patients, time to acquire data, reduced signal quality and artifacts, costs and the vulnerability of the target group [8].

BCI research aiming at bringing BCI to end-users at home faces a translational gap that refers to the lack of detailed knowledge about the end-users of brain-computer interfacing and bio-psycho-social facets of this human-computer interaction [9]. Such knowledge is mandatory to successfully transfer BCI developments from the laboratory of developers to the end-users in need [10], [11].

### The framework of the User-Centered Design

The user-centered design (UCD) focuses on usability, i.e. how well a specific technology suits its purpose and meets the needs and requirements of the targeted users and was standardized in the ISO 9241–210 [12]. The six principles of this approach are listed in Table 1 and include early and continuous involvement of potential users; understanding of user requirements and the whole user experience; and iterative processes between developers and users. To implement these principles in the iterative process of assistive technology (AT) development [13], four practical stages were defined, which address understanding and specification of users' needs and the context of use, and evaluation against the defined requirements (see Table 1). The ISO 9241–210 defines usability as the “extent to which a […] product […] can be used by specified users to achieve specified goals with effectiveness, efficiency and satisfaction in a specified context of use” (page 3). This definition of usability implies that a BCI-controlled application cannot be evaluated without taking into account the context of its use, and constitutes, thus, a holistic approach to the user experience [14], [15].

**Table 1:** Principles and stages of the user-centered design (left column) and their transfer to BCI-controlled applications (right column).

The Principles (P)	BCI-controlled application
P1: understand the user, the task and environmental requirements	Chose appropriate metrics - apply questionnaires for first definition [59]
P2: encourage early and active involvement of users	Interaction between users and developers to define the first version of a prototype [50]
P3: be driven and refined by user-centred evaluation	Valid evaluation metrics [22]
P4: include iteration of design solutions	Continuous interaction between developers and end-users in their home environment leading to several prototypes [21], [51], [60]
P5: address the whole user experience	Evaluation metrics that covers all aspects of “usability”, i.e. effectiveness, efficiency, satisfaction [21]
P6: encourage multi-disciplinary design	BCI team of computer scientists, engineers, psychologists, medical doctors, neuroscientists, AT experts
**The Stages (S)**	
S1: understand and specify the context of use	Identified need and potential impact [50], [51]
S2: specify the user requirements	Questionnaires and interviews [51], [59]
S3: produce design solutions to meet user requirements	Prototypes available for testing [22], [33], [35], [50], [51]
S4: evaluate the designs against requirements	Evaluation metrics (effectiveness, efficiency, satisfaction) [60]

This iterative approach has been realized with the BCI controlled Brain Painting. Numbers in parentheses refer to the publication in which the corresponding steps were realized.

Effectiveness refers to how accurate and complete users can accomplish the task at hand. Efficiency relates the invested costs to effectiveness, i.e., the users' invested costs and time. User satisfaction entails the perceived comfort and acceptability while using the product. We define the “product” as the *BCI* controlled application. The context of use refers to users, tasks to be accomplished, equipment, i.e. hardware, software, and materials, and the physical and social environments in which a product is used ([12], page 2). Importantly, participants in a UCD process should be chosen to match the expected user population as close as possible, thus, clearly implying the involvement of motor impaired individuals in the evaluation of BCI controlled applications. For evaluating AT prototypes, end-users need to experience those as imagining such experience does not suffice and may be impossible for the end-user [16]. Further, the evaluation tasks should be representative for most users such that results can be generalized beyond the specific sample. While such tasks have already been used for the evaluation of single or few aspects of the BCI-end-user interaction, usability with all its facets, as described above, has not yet been addressed with a larger sample of end-users with severe impairment including the locked-in state and with applications aiming at different aspects of daily living, namely communication and entertainment. For example, Lorenz and colleagues investigated several usability aspects such as accuracy, workload and learnability with regards to hybrid BCIs in a sample of 12 healthy participants [14]. Likewise, Pasqualotto and colleagues compared accuracy, usability, and workload in two BCIs controlled by different input signals, namely slow cortical and event-related potentials [17], [18]. In contrast, McCane and colleagues included a large sample of N = 25 end-users with amyotrophic lateral sclerosis in all stages of the disease, but evaluated only accuracy [19].

We operationalised all three aspects of usability – effectiveness, efficiency and satisfaction - to allow for evaluation of BCI controlled applications and thus, introducing the user-centered approach in BCI development [20]–[22]. The aim was to provide a framework for an evaluation based on standardized, generic metrics that can be applied irrespective of the specific location of the research team, the specific end-user, the input signal, and the application. The present paper reports on experience of a unique sample of severely motor impaired end-users of BCI, and allows, thus, cautious conclusions for usability. Subsets of these data were previously published as indicated.

## Methods

### Evaluation metrics

Following the definition of usability, we introduce metrics for effectiveness, efficiency, and satisfaction (see Table 2 for all evaluation metrics).

**Table 2:** Evaluation metrics for each aspect of usability.

Aspects of usability	Transfer to BCI/applications	Metrics	Assessment
Effectiveness	Accuracy	% correct responses	each session
Efficiency	Information transfer rate	Bits/min	each session
	Utility metric	Bits/min (bits/min = 0 if effectiveness <50%)	each session
	Workload	NASA-TLX	each session/task
Satisfaction	General aspects of AT	QUEST 2.0	end of prototype testing
	BCI related aspects	4 items (reliability, learnability, speed, aesthetic design)	end of prototype testing
	Match between product and user	ATD-PA Device-Initial, Sections Consumer, Professional	end of prototype testing
	Overall satisfaction	VAS (0–10)	each session
	Interview	Semi-structured	end of prototype testing
	Use in daily life	Single item	end of prototype testing

NASA-TLX  =  NASA Task Load Index.

QUEST  =  Quebec User Evaluation of Satisfaction with Assistive Technology.

ATD-PA  =  Assistive Technology Device Predisposition Assessment.

VAS  =  visual analogue scale.

#### Effectiveness

A measure of how accurate and complete a user can accomplish a BCI-controlled application is how often the intended output can be achieved. Accuracy relates successful selections to the total number of attempted selections and can be expressed in percentage of correct responses.

#### Efficiency

Efficiency relates the costs, i.e. effort and time, invested by the user to effectiveness. An objective measure of efficiency is the information transfer rate (ITR) and its modifications with regards to error probability, accuracy, and practicality while workload constitutes a subjective measure.

Information transfer rate: The ITR, which takes into account the available number of possible selections and the time needed for a selection, serves as an objective measure of efficiency for applications aiming at communication (in the broadest sense). It is expressed in bits per minute and incorporates speed and accuracy in a single value. A common phenomenon in a BCI-controlled application is that high ITR can be achieved despite numerous miss-selections if the number of possible selections is high. However, such a BCI would be of no practical value as no meaningful communication would be possible. Thus, the utility metric was introduced and takes into account, that with an accuracy below 50%, no reliable communication can be achieved, i.e. ITR is 0 bits/minute for all accuracies below 50% [23]. Different ways to obtain ITR have been suggested and discussed in the literature [24].

Workload: For the assessment of subjective workload the NASA Task Load Index (TLX) was chosen [25]. Workload in the NASA TLX is defined as a “hypothetical construct that represents the cost incurred by a human operator to achieve a particular level of performance” (p.140). The NASA TLX measures the overall workload experienced while operating a specific application and identifies main sources of workload which is estimated across the dimensions mental, physical, and temporal demand, and performance, effort, and frustration. Subjective workload for each dimension has to be rated on twenty step bipolar scales with scores from 0 to 100. A weighting procedure combines the individual scores for each dimension into one total score. It has previously been used to assess workload of healthy subjects during BCI operation [14], [18].

#### Satisfaction

User satisfaction refers to the perceived comfort and acceptability while using the product. We suggest several measures to assess device satisfaction.

Satisfaction with general aspects of the device: The Quest 2.0, the Quebec User Evaluation of Satisfaction with assistive technology (QUEST 2.0, [26]), allows for quantifying satisfaction with general aspects of a product and was previously suggested for the measurement of usability of AT [13]. The questionnaire consists of items that cover 12 aspects. The QUEST 2.0 is considered invalid if scores for more than six (of 12) satisfaction items are missing; thus, it is important to check whether the items are adequate to assess a specific application. We considered the items “durability, service delivery, repairs/servicing, and follow-up services” inadequate for the evaluation of a BCI-controlled application during development and removed those from the questionnaire. “Durability” was removed also because EEG amplifiers have already demonstrated their long-term functionality, electrodes have to be replaced depending on the frequency of use, and our evaluation procedure did not span a time frame of years such that durability could become an issue. Items are rated on a Likert-type scale from 1 to 5. Whenever users are not “very satisfied” they are invited to comment. The arithmetic mean across all items provides the total satisfaction score.

BCI specific items: Demers and colleagues explicitly invite researchers to add few items to render the questionnaire more suitable for a specific piece of technology [26]. Thus, to render the QUEST 2.0 more suitable for evaluation of BCI controlled AT we added the four items reliability, speed, learnability, and aesthetic design. With “reliability” we refer to how reliable the EEG signal can control the BCI within and between sessions, “learnability” refers to the time and effort needed to learn how to control the BCI including aspects such as learning to modulate the respective brain response and the functions of the application. The content of all items were made explicit to the end-users. This BCI adapted QUEST is referred to as “Extended QUEST 2.0.” [22]. The added items cannot be integrated in the total score of the QUEST [26]. As the added items are particularly relevant for BCI development, we recommend reporting scores for each item in addition to a total score. To ensure content validity, the work of Batavia and Hammer (1990) and Scherer and Lane (1997) who developed consumer-based criteria for evaluation of AT in focus-groups of AT users, and the experience of BCI and AT experts were used as sources for item selection [27], [28]. Finally, users can be asked to indicate the three most important items of the Extended QUEST 2.0.

Overall satisfaction: The Extended QUEST 2.0 is not suitable to be applied after every BCI session as it requires time and it is unlikely that basic aspects contributing to satisfaction change substantially across sessions with the same BCI-controlled application. However, we consider it valuable to obtain a coarse rating of overall satisfaction at the end of each BCI session. Visual analogue scales (VAS) can provide such a measure. Thus, users can be easily asked after each session to indicate their overall satisfaction on a VAS ranging from 0 (not at all satisfied) to 10 (maximally satisfied). Such a rating does not provide any in depth information about the sources of satisfaction/dissatisfaction, but it allows for easy monitoring specifically in long-term studies [29].

#### Use in daily life

An important aspect for any BCI-controlled application is whether a potential user of the device can imagine indeed using the application in daily life. We argue that the better the match between potential end-user's needs and the possibilities offered by the AT, the more likely it is that the application will finally play a role in daily life of users. We suggest a respective questionnaire and a face valid question to assess this aspect.

Match between AT and the user: The Assistive Technology Device Predisposition Assessment (ATD PA) is part of the set of questionnaires according to the Matching Person and Technology Model (MPT) [30]. It has been previously suggested for evaluation of prototypes [13] and we used the questionnaire ATD PA Device Form – Initial Consumer and Professional. It addresses the expected technology benefit by asking the end-users (Section Consumer) and the professional users/AT experts (or the researchers) (Section Professional) to rate their predisposition of the consumer for using the AT under consideration. The 12 items of the ATD PA Section Consumer Form have to be rated on a 5-point Likert-scale from 1 to 5. Users have the option to indicate a “0” if the item is not applicable. The arithmetic mean provides a total score. The highest possible score is 5.0. Scores between 4.0 and 5.0 indicate a good match of person and AT device. Scores below 4.0 indicate that the match could be improved. If an item is scored 3 or less, there is a risk of device non-use [30].

Single question about use in daily life: The ultimate proof of BCI use in daily life is its actual use. To date, the closest we can get to information about potential use in daily life is a face valid question: “Based on your experience with the BCI-controlled application: Can you imagine using the BCI for communication/entertainment in daily life”? It has been suggested that a single overall opinion may be a good indicator for overall evaluation results [16].

#### Application specific metrics

BCI-controlled applications differ considerably and thus, to receive more application specific details any face valid measure can be applied in addition to the proposed evaluation metrics. For example, for the Brain Painting application visual-analogue scales were introduced to assess frustration and joy and were applied after every session [29], [31].

### End-user Sample

Four different prototypes were tested by N = 19 participants with severe motor impairment; n = 15 tested one, n = 2 two and n = 2 three prototypes. These potential end-users of BCI technology were either BCI novices or had some experience with BCI due to being involved in previous studies. All users had experience with AT in their daily lives and thus, had an adequate standard to which the BCI-controlled application could be compared to. Table 3 describes the end-user sample.

**Table 3:** Demographic, disease and AT/BCI related data of end-users; speaking not possible unless mentioned.

EndUser	Age	Diagnosis	Artificial ventilation	Artificial nutrition (PEG)	Wheel-chair	Residual muscular control	Computer input device	BCI-prototype	Level of impairment^1^
**1**	55	Amyotrophic Lateral Sclerosis	No	No	Yes	Eye movement, mimic, movement of the head, speech	Chin joystick/switch and screen keyboard	Spelling, Brain Painting	3
**2**	50	Spinal Muscular Atrophy Type III	No	No	Yes	Eye movement mimic, movement of the head, residual movement of fingers, speech	Mouse plus screen keyboard, speech recognition	Spelling	2
**3**	39	Muscular Dystrophy Duchenne	Yes 24 hours non-invasive	Yes	Yes	Eye movement, mimic, restricted speech due to ventilation	Chin joystick and Wergen keyboard	Spelling, Brain Painting, Spelling-hybrid	3
**4**	37	Muscular Dystrophy Duchenne	Yes, non-invasive, 24 hours	Yes	Yes	Eye movement, mimic, minimal residual movement of fingers, restricted speech due to reduced muscular strength	Joystick (AT) and screen keyboard	Spelling	3
**5**	54	Amyotrophic lateral sclerosis	Yes, non-invasive, some hours per day	No	Yes	Eye movement, mimic, movement of the head and shoulders, restricted speech	Head tracker	Brain Painting	3
**6**	45	Stroke (pontine infarction due to basilar artery thrombosis)	No	No	Yes	Eye movement, mimic, movement of head and shoulders, residual movement of fingers, restricted speech due to restricted respiration (voice amplifier)	mouse and screen keyboard	Brain Painting, Connect-4, Spelling-hybrid	2
**7**	48	Hemiplegia after cerebral bleeding (brain stem aneurysm)	No	No	Yes	Eye-movement, speech, residual movement of left arm, hand and head, mimic	keyboard	Connect-4	2
**8**	45	Infantile cerebral palsy	No	No	Yes	Eye movement (unreliable), mimic, residual movement of hand/arm	Switch with arm, letter board with eye-movement	Connect-4	4
**9**	45	Tetraparesis after cerebral bleeding in basal ganglia (right frontal-temporal lesion)	No	Yes	Yes	Eye-movement (unreliable), mimic, residual movement of one finger of left hand (depending on physical state)	Button by finger press (yes/no)	Connect-4	4
**10**	26	Spinal muscular atrophy Type II	No	Yes	Yes	eyes, mimic, head, speech	normal mouse and virtual keyboard	Spelling-hybrid	2
**11**	52	Amyotrophic lateral sclerosis	No	No	Yes	eyes, mimic, head, strong restricted speech (caregiver translates)	Slowed keyboard	Spelling-hybrid	3
**12**	40	Hemorragic stroke	no	no	yes	legs, hands and shoulder with marked spasticity, residual movements of head and face mimic	keyboard	Spelling	3
**13**	47	Amyotrophic lateral sclerosis	no	no	yes	head, mimic	no	Spelling	3
**14**	48	Ischemic stroke	no	no	yes	head, mimic	eyetracker	Spelling, Spelling-hybrid	3
**15**	21	Hemorragic stroke	no	no	yes	upperarms, head, mimic	no	Spelling	3
**16**	43	Spinal cord injury	no	no	yes	paraplegic	mouse	Spelling-hybrid	2
**17**	54	Hemorragic stroke	no	no	yes	arm and head, speech	keyboard and mouse	Spelling-hybrid	2
**18**	49	Amyotrophic lateral sclerosis	no	no	yes	weak arm and head, speech	mouse	Spelling-hybrid	2
**19**	23	Spinal cord injury	no	no	yes	paraplegic	keyboard and mouse	Spelling-hybrid	2

1 The numbers for level of impairment are according to Kübler and Birbaumer (2008): 1 =  minor impairment: slightly impaired limb movement, normal speech; 2 =  moderate impairment: restricted limb movement (wheelchair) and unaffected speech OR intact limb movement, but no speech; 3 =  major impairment: almost tetraplegic, restricted speech; 4 =  locked-in state: only few muscles for communication (e.g., eye movement), no speech.

#### Ethics Statement

All studies were conducted in accordance with the latest version of the Declaration of Helsinki (October 2013; http://www.wma.net/en/30publications/10policies/b3/) and approved by the Ethical Review Boards of the Medical Faculty, University of Tübingen and Fondazione Santa Lucia. All participants were informed in detail about the study and signed informed consent.

### Input signals for BCI

Either event-related potentials (P300-/ERP-BCI) or sensorimotor rhythms (SMR-BCI) were used as input signal for BCI control.

For the detailed explanation of signal acquisition and data analysis we refer to the original studies [21], [22], [32]–[36]. The EEG was recorded with 8 or 16 channel caps (see Table 4).

**Table 4:** Summary of applications and number of patients involved.

Application	Input signal for BCI	Domain	Number of patients involved	Number of channels	Number of sessions with BCI
Spelling with comer-cial AT software (Qualilife) [22]	P300	communication	8	8	4
Brain Painting [21]	P300	entertainment	4	16	7
Spelling with AT software – hybrid [35], [47]	P300 + EMG^1^	communication	9	8	1
Connect 4 [33]	SMR^2^	entertainment	4	16 (64)	6

1 electromyogram;

2 sensorimotor rhythms.

The main difference between the two input signals is that event-related potentials are triggered in the brain by external stimulation, typically in an oddball-paradigm (e.g., [37] for review), while sensorimotor rhythms have to be actively modulated by the user, who is usually instructed to imagine a movement with finger, hands, arms or feet (motor imagery) [3], [38]–[40]. Depending on the input signal BCIs were referred to as “reactive”, because the brain reacts to stimulation, and “active” because a specific state has to be actively evoked by the end-user [41].

In both, P300-BCI and SMR-BCI effectiveness (accuracy) was expressed as % correct responses. Due to the number of trials available, efficiency in terms of ITR could not be calculated according to Nykopp [42] and was thus, always reported according to Wolpaw and colleagues [43]. Zickler and colleagues also included the utility metric [23].

**Figure 1:**
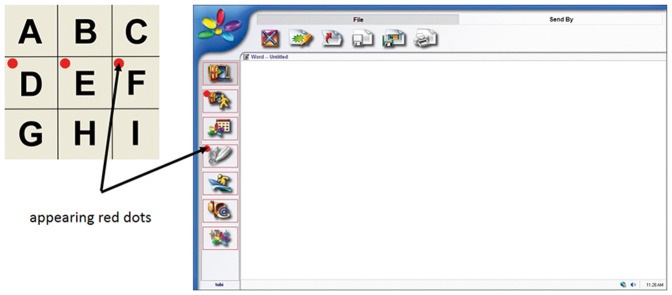
Transfer of the matrix based speller paradigm to the Qualilife software. Left: To adapt end-users to the flashing of dots, those were placed in each cell of the well familiar matrix. Instead of the letters those dots were flashed. Right: Screen shot of the Qualilife communication application. The now familiar red dots were assigned to each option of the Qualilife communication and control surface. Red dots appear randomly at each possible “button” to press. Attention needs to be focused on the specific button to be pressed by counting how often the red dot is appearing.

For communication the P300-BCI approach was implemented into a commercially available AT Software (QualiWORLD by QualiLife SA, Lugano, Switzerland). In this first prototype, the visual stimulation to elicit ERPs deviated from the classic P300 speller in which letters were flashed row- and columnwise. Instead, red dots were allocated to each cell in the matrix and the end-users' task was to count how often the red dot appeared [22], [44]. Those red dots were also allocated to “buttons” and links in an emailing program and internet browser. The red dots appeared in random order and the users' task was to count how often the red dot appeared besides the to-be-selected item (Fig. 1). After selection of a specific dot, the respective link was followed and a new page was opened. As the number of averages to achieve good performance was still high and due to feedback of end-users who stated that the red dot would be too “flashy”, in the second prototype different stimuli (red and green dots and grids) were implemented and chosen individually for each end-user [32], [35]. Other stimuli were also available, could be chosen individually, and more can be easily added.

### Applications

Four different applications were tested by end-users: two for communication and two for entertainment (Table 4).

#### Communication

With the first prototype that implemented the P300-BCI into AT software it was possible to enter text (text entry), to write and send electronic mails (emailing) and to surf the internet (browsing). The prototype was evaluated by 8 healthy participants within one [44] and eight end-users with severe motor impairment within 4 sessions (4 end-users in [22] and 4 not previously published). Feedback of participants led to the second prototype which integrated the hybrid concept [45], [46]. With an EMG controlled switch it was accounted for low speed and the lack of a delete option [34], [47], [48] (see [35], for full description of the prototype). Tasks to be completed were also text entry and emailing. In both prototype evaluations, text entry had to be completed in the copy spelling and free spelling mode [49]. Nine end-users were included in the evaluation procedure (Table 4) (3 published in [35], 6 unpublished).

#### Entertainment

**Figure 2:**
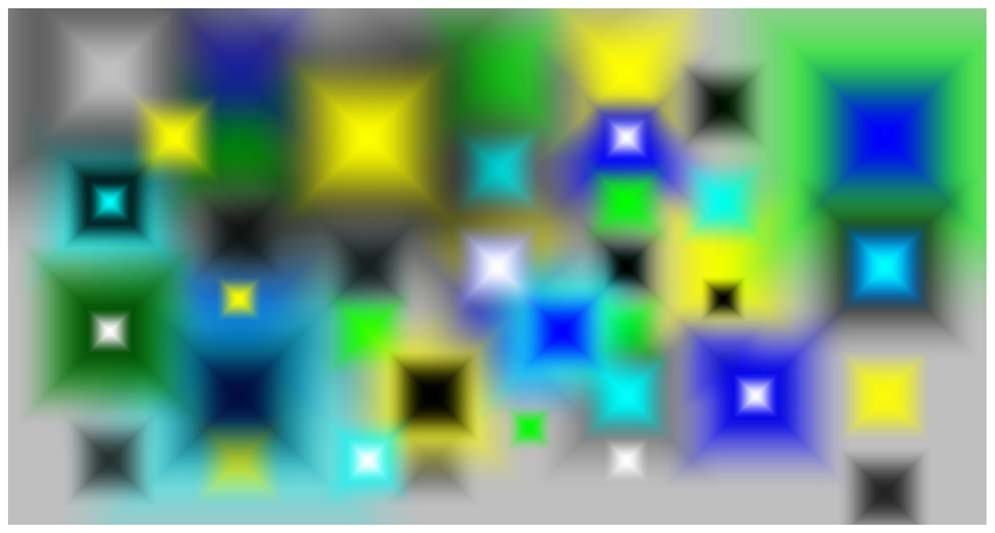
“Grau-Gelb” (engl. “grey-yellow”) - a painting created with the Brain Painting application by a locked-in end user with amyotrophic lateral sclerosis (© J Thiele, with permission).

Brain Painting: The Brain Painting prototype was evaluated in seven sessions, five of which in the free painting mode, by four severely impaired end-users [21] (see Table 4). Figure 2 depicts a painting by an end-user. The iterative user-centered approach for refining the P300-BCI controlled Brain Painting application is summarized in Table 1. In the Brain Painting application letters of the classic P300 matrix are replaced by icons representing cursor position on the virtual canvas, objects (square, circle, cloud), opacity, zoom in/out, color, and backspace for correction of unintended selections. Choosing the color places the object on the virtual canvas. Thus, several selections are necessary before an object appears on the canvas [21], [29], [50], [51].

Connect 4: The well known Connect 4 game was adapted such that it could be controlled by an SMR-BCI. Connect 4 is a strategic game for two people who play against each other. Coins are placed in rows and columns with the goal to connect four coins in a row or column before the opponent can do so; the first to succeed wins the game. The game realizes a 2-class motor imagery (MI) paradigm and the end-user can select a row by moving the upper cursor from left to right or right to left (depending on the MI class, e.g. left hand or right hand) and place a coin by moving the cursor downward (e.g., by feet MI). The MI classes were individually determined in a calibration session prior to BCI use for gaming. The prototype was evaluated by four severely impaired end-users in a copy and a free mode [33], [52].

## Results

All aspects of evaluation for applications covering communication and entertainment could be performed with severely motor impaired users including those in the locked-in state. Published results for end-users and prototypes can be found in [21], [22], [32]–[36]. Here, we report the mean and range for the respective measure and application in order to demonstrate the applicability of the evaluation metrics (see Tables 5 and 6 for all results).

**Table 5:** Summary of evaluation results.

	Effective-ness	Efficiency ITR	Satis-faction; VAS	Satis-faction; QUEST; BCI specific	Satis-faction; ATD PA	Use in daily life
Applica-tion	mean [%], range [%]	mean, range	mean, range	mean, range	mean, range	(yes/total)
Spelling^1^	81.3	5.7	6.9	3.8; 1–5	-	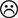
	57.1–100	2.6–8.6	3.5–10.0	3.5; 1–5		(1^7^/8)
Brain	89.3,	4.9	6.7	4.2; 3–5	3.9	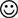
Painting^2^	86–93	4.6–5.2	5.0–7.9	4.4; 2–5	3.4–4.3	(3/4)
		4.7^3^				
		4.3–5.2^3^				
Spelling	87.9	11.9	7.7	3.7; 1–5	2.7	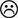
hybrid^4^	78–100	2.6–36.6^5^	5.0–10.0	3.7; 1–5	1.3–3.4	(1/4)^8^
Connect 4^6^	60.0	0.53	7.7	3.8; 2–5	3.3	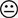
	40–80	0.1–1.4	2–10.0	3.9; 2–5	2.3–4.3	(2/4)

1 4 sessions (copy spelling, free spelling, emailing, internet surfing).

2 data refer to the last of 5 free painting sessions.

3 Utility metric.

4 3 sessions (copy spelling with and without EMG correction, free spelling (sentence) and emailing).

5 ITR for BCI only; EMG correction not included.

6 6 sessions (screening, copy task and free mode playing).

7 the end-user stated “maybe”.

8 only 4 of 9 end-users were asked this question.

**Table 6:** Mean and range for all dimensions of workload and total score for each application averaged across tasks and end-users.

Application	mental demand	physical demand	temporal demand	performance	effort	frust-ration	total score
**Spelling**	12.2	6.7	5.8	4.3	10.1	2.2	41.9
	1–25	0–33	0–20	0–17	1–27	0–15	9–77
**Brain**	7.0	4.8	7.0	5.0	6.3	2.3	31.5
**Painting**	2–20	0–17	1–17	1–8	2–16	0–8	21–49
**Spelling**	11.5	5.3	6.4	3.0	8.3	3.0	39
**hybrid**	0–32	0–30	0–27	0–10	0–21	0–27	12–72
**Connect 4**	10.7	5.0	9.5	3.4	5	4.8	37.5
	0–30	0–24	0–27	0–10	1–6	0–27	17–72

The possible range of each subscale and the total score is 0 to 100.

### Effectiveness

With the P300-/ERP-BCI end-users achieved an accuracy of up to 100% and were on average in the range of possible meaningful communication which requires an accuracy of at least 70% [49]. Average performance with the SMR-BCI was 60%, varied between 40 and 80% and was thus below that of the P300-BCI. For both BCI input signals across all applications, performance varied between sessions and end-users (range 40–100).

### Efficiency

Information Transfer Rate:_Similar to accuracy ITR (according to [43]) varied between sessions and end-users. For the Brain Painting application the utility metric was also calculated, but as all subjects performed well above chance level, both metrics provided similar results. The hybrid approach to BCI provided highest ITR, as here the number of sequences was adapted individually whereas in the other application a fixed number of sequences was used. ITR during spelling was higher than for Brain Painting. This was due to longer pause intervals between item selections to provide end-users with sufficient time to think about what to select next for painting. The P300-BCI provided considerably higher ITR than the SMR-BCI application (Table 5).

Workload: The P300-BCI controlled painting application imposed the lowest total workload on the users (Table 6). Specifically, mental demand was lower as compared to all other applications. Effort was lowest in the SMR-BCI controlled Connect 4 gaming application, which also elicited the highest temporal demand and frustration. Table 6 presents the detailed results for each workload dimension and application.

### Satisfaction

Satisfaction with general aspects of the device: The range for all applications was between 1 and 5 and average ratings were 3.7. With ratings above 4, the participants indicated higher satisfaction for the Brain Painting application as compared to spelling and SMR controlled gaming.

**Figure 3:**
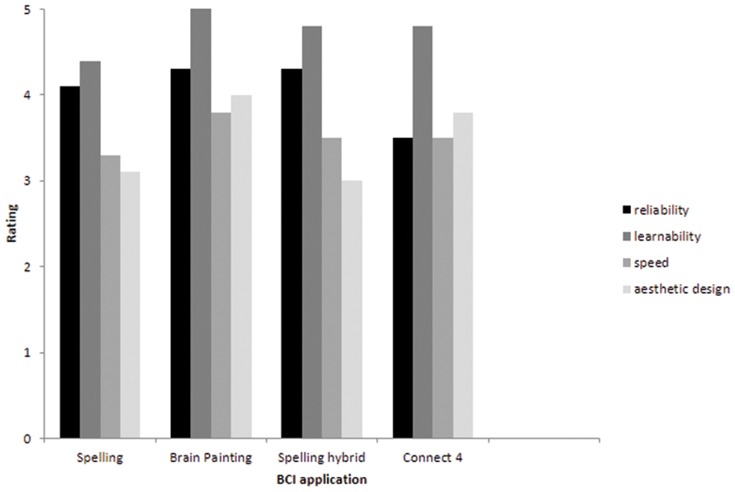
Ratings for the BCI specific items (explanation see text).

BCI specific items: These items cover reliability, learnability, speed and aesthetic design. As those are particularly relevant for BCI development, we report those in more detail (Fig. 3). Satisfaction with reliability was rated high (above 4 of 5) for all P300-BCI applications and below 4 for the SMR-BCI application Connect 4. Learnability was highly satisfactory for all applications. With around 3, speed was rated moderate, with the P300-BCI applications not superior to the SMR-BCI application. Aesthetic design of the AT altogether (including the electrode cap) was rated between 3 and 4 and was rated lower for the communication as compared to the entertainment applications.

Overall satisfaction: Overall satisfaction ratings ranged from 2 to 10 and thus, covered almost the entire range.

### Use in daily life

Match between AT and the user: The ATD PA, Device – Initial, Section Consumer was available for the P300-BCI Brain Painting application, the Hybrid Prototype for Spelling and the SMR-BCI Connect 4. The range for Brain Painting was between 3.4 and 4.3 indicating a good match for some users and room for improvement for others. For Connect 4 the lowest rating was 2.3 and for the hybrid prototype 1.3 which implies risk of non-use.

Single question for use in daily life: The single question about whether the potential end-users of BCI-controlled applications could imagine using the device in daily life was answered by all but 5 subjects (spelling hybrid). For the two spelling applications the answer was “No” by all but one subject. For Brain Painting 3 end-users answered “Yes” and one “No”, and for Connect 4 the answer was “Yes” for two end-users, “Yes, if it worked better” for one and “No” for one.

Comments by end-users: At the end of prototype testing, end-users were asked to comment. Likewise, for satisfaction ratings below “very satisfied” end-users were asked for their reasons of dissatisfaction. All end-users provided comments. Those in the locked-in state prepared their comments ahead of time or after the final evaluation.

For all applications the set-up of hard- and software and specifically the electrode cap with gel was identified as the main obstacle for regular use in daily life. Exemplary statements were “*Adjustment of EEG-cap and electrodes is too cumbersome*”, “*I would be very satisfied if everything could be smaller, e.g. compressed in one device*”, “*cap looks too much like a device used in hospital*”, “*The BCI-application is good in general, but everything takes too long, e.g. set-up of BCI and the motor imagery training*” (Connect 4, three users); “*Should be smaller, have less parts*”, “*less electrodes and no cables would be better*” and [with regards to cables] “I *cannot change sitting position in the wheelchair or move around; in the public I would feel a bit uncomfortable as the device is so big and eye-catching*” (Brain Painting, three users); “*looks strange to have many cables and electronic stuff at one's body*” (Spelling hybrid, two users); “*one needs assistance, but not too difficult*” (Spelling hybrid, one user); “*the more you use it, the easier it gets*” (Spelling hybrid, one user); “*it takes a while to adjust the EMG, but when it works, it is cool*” (Spelling hybrid, one user); “*too many different parts that have to be attuned to each other*”, “*Preparation takes too much time”*, and „*very technical*“ (Spelling, three users); “*too cumbersome, would not be able to take it with me*” (Spelling one user).

Further, speed was judged as too slow as compared to conventional AT software which was used by all participants for communication and interaction in daily life. Exemplary comments were “*five times faster would be acceptable*” and “*eye tracking systems allow faster selections*” (Brain Painting, two users); “*takes too long for real communication or for writing longer sentences/text*” (Spelling hybrid, one user); the BCI should be “*twice as fast*”, “*three to four times faster*”, and “*with my own AT I can write 90 characters per minute*”(Spelling, three users); “*stimulation tiring*” (Spelling, two users);“*stimulation too fast*” (Spelling one user); “*too slow, should be faster*”, “*it did not work in my case”* (Connect 4, two users).

## Discussion

The here suggested evaluation procedure can be applied to potential end-users of BCI-controlled applications in the field [22], [29], [33], [35], [38], [53], [54] and guide the development and further refinement of BCI. The concept of usability proved adequate to elicit valuable information that can be fed into the iterative process between users and developers as suggested by the UCD. The samples of end-users included in the studies also comprised end-users in the locked-in state who have only residual muscular movement, most likely eye movement. The here suggested evaluation metrics could also be applied successfully to this end-user group which is most often seen as target population for BCI-controlled AT devices (see e.g., [55]). We, thus, successfully transferred the UCD to BCI research and development, and it provides a framework for standardized evaluation of BCI controlled applications.

Studies included here, provided applications for communication in a broad sense. However, other BCI applications exist, e.g., for rehabilitation after stroke [56], [57] and restoration of limb movement after high spinal cord injury [53], [58]. The suggested evaluation metrics are also suitable for such applications albeit the weight of each usability component may differ. For example, for stroke rehabilitation the task at hand to complete (effectiveness) could be to produce the required brain activity patterns or to move an orthosis. Brain activity patterns as a measure of effectiveness would not have to be produced as reliable and accurate as necessary for communication. However, if the movement of an orthosis serves as a measure of effectiveness the reliable operation may be much more important for successful outcome as it provides direct feedback to the end-user of his or her performance and too many failed attempts may discourage users.

The number of correct selections was on average above 80% for all P300-BCI based applications indicating high effectiveness. With 60% the SMR-BCI based gaming application was clearly below that performance which corroborates that BCIs using event-related potentials as input signal are more practical for communication as they more likely fulfill the end-users' wish for higher communication speed as currently provided by SMR-BCI. A result that is also supported by Lorenz and colleagues with healthy participants who were slower and less accurate when item selection had to be performed with motor imagery and confirmed with event-related potentials (ERP) as compared to vice versa or solely ERP [14].

With regards to efficiency, information transfer rates were considerably higher for the P300-BCI controlled applications. The hybrid prototype for spelling provided an ITR twice as high as the classic P300-BCI. Hybrid approaches to BCI became more popular in the past few years as they advantageously take into account any physiological response available to the end-user [14], [45], [46]. The total workload was moderate for all BCIs with a broad range. It is important to note that objective and subjective measures of efficiency may considerably dissociate. For the BCI controlled Connect 4 application ITR was low, but subjectively rated workload was on average in the range of the P300 controlled applications. Thus, ITR alone is not a valuable indicator of the potential usability of the targeted application. This result is important as it is often referred to the ITR when arguing that one type of BCI outperforms another. The calculation of ITR has to be carefully chosen because high an ITR may not necessarily correspond to meaningful communication.

Average satisfaction as measured with the Quest 2.0 was equal or above 3.7 (rating scale from 1 to 5) for all applications and high for the Brain Painting application. Importantly, satisfaction with the SMR-BCI controlled gaming application was equal to that of the P300-BCI controlled spelling applications despite considerably lower effectiveness and efficiency (ITR) which might reflect higher error tolerance when using a BCI for entertainment. This again corroborates the need of multilevel assessment when aiming at bringing BCI-controlled applications to end-users. Ratings for the BCI specific items were above or equal to 3.5 for all applications. End-users rated learnability for all BCI applications high. Likewise, reliability was rated high for all P300 applications including the hybrid BCI. Satisfaction with aesthetic design was moderate, and care has to be taken that the device does not attract even more attention to end-users with disability.

None of the end-users involved in our evaluation studies could imagine using the BCI spelling applications for communication in daily life, as assessed with the face valid question. In contrast to the spelling applications, 5 of 8 end-users could imagine using those for entertainment in daily life. Surprisingly, this held also true for the SMR-BCI controlled Connect 4 despite lower ratings for reliability and only low effectiveness and efficiency (ITR). Also, lower ratings for aesthetic design did not affect the vision of daily BCI use. These results suggest that potential end-users of BCI-controlled applications are more tolerant with regards to reliability, speed, and aesthetic design when the BCI is aiming at entertainment as compared to communication. In contrast to communication, entertainment – here: gaming and painting – is for joy and pleasure and can be regarded as an add-on provided communication is ensured. This higher error tolerance was corroborated by an end-user with ALS who has been using the Brain Painting application in daily life without experts present. She indicated high satisfaction despite frequent low to moderate subjectively perceived BCI control [29]. However, when it comes down to the basic need of communication, obstacles are less tolerated as only one of 12 potential end-users could imagine using the BCI for communication in daily life. This is an important result for BCI developers as it clearly demonstrates that if BCI-controlled applications are aiming at communication, effectiveness and efficiency are of highest importance, whereas when entertainment is the goal, it might be more focused on design and other gadgets. However, this assumption needs to be confirmed in future studies. Other face valid measures of BCI usability in daily life are the number and duration of BCI sessions [29], [31].

## Conclusions

**Figure 4:**
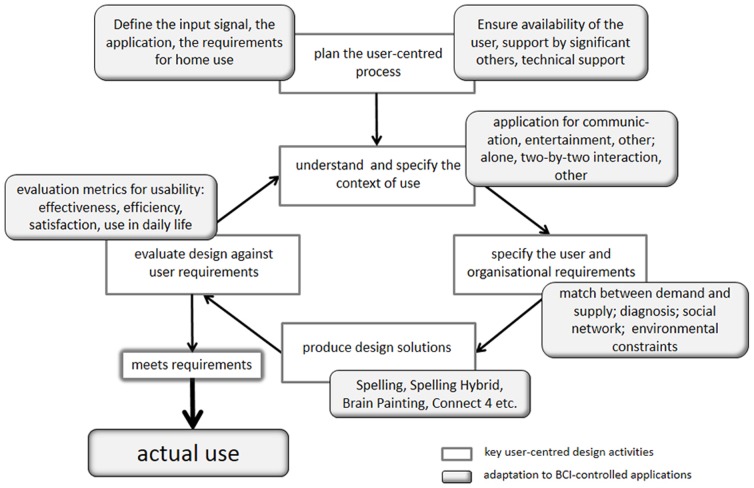
Key user-centered design activities (from [12]) adapted to BCI-controlled applications. If the application matches the individual end-user's needs, it will very likely be used in daily life.

The UCD to the development of computer-based interactive systems provides a theoretical framework which can guide the design of mandatory translational studies on how to transfer BCI-controlled applications from the laboratory of developers to the end-users' homes (Fig. 4). Appropriate measures for evaluation of usability are now available and proved to be deployable with severely paralyzed and locked-in potential end-users of BCI-controlled applications. This is an important result as such end-users may be restricted in their attentional capacities and their time available for such evaluation. In addition to these basic measures of usability, which include effectiveness, efficiency and satisfaction, application specific metrics can be added to either category and open interviews can provide more detailed information. Thus, the UCD appears to be suitable as a solid pillar for bridging the translational gap. Evaluation results of the here summarized studies suggest that applications for communication and control require higher accuracies to be perceived satisfactory than those for entertainment and that if the BCI-controlled application matches the end-users needs it is used despite low to moderate effectiveness. If BCI developers are willing to participate in the iterative process of the UCD and to take its results into account, we are more likely to provide BCIs that match the end-users needs and will be used in their daily life. We are confident that with further evaluation studies along the UCD, the BCI community will eventually be able to provide indication criteria for individual users and the type of BCI, and to establish home use without experts being present.
